# Trafficking Unconventionally via UPS

**DOI:** 10.3390/cells9092009

**Published:** 2020-09-01

**Authors:** Bor Luen Tang

**Affiliations:** Department of Biochemistry, Yong Loo Lin School of Medicine, National University of Singapore, Singapore 117596, Singapore; bchtbl@nus.edu.sg; Tel.: +65-6516-1040

Conventional protein secretion in eukaryotic cells occurs via vesicular trafficking of proteins that are first targeted to the endoplasmic reticulum (ER), through the Golgi apparatus, and subsequently routed to the plasma membrane (PM), where membrane proteins take up residence while luminal proteins are released extracellularly. However, a good number of cytoplasmic and membrane proteins are found to be secreted or PM-transported either alternatively, or exclusively, via an unconventional or non-canonical mode or route [1,2,3]. Accordingly, that a protein undergoes unconventional protein secretion (UPS) is recognized by a lack of its initial ER targeting, its bypassing of the Golgi (or the need for an intact Golgi apparatus), or documented insensitivity of the transport to classical inhibitors of conventional exocytosis (such as brefeldin-A). Prominent examples of unconventionally secreted proteins in animal cells include both soluble cytosolic proteins such as fibroblast growth factor 2 (FGF2), interleukin-1β (IL-1β), and galectins, as well as membrane proteins such as α-integrin and the cystic fibrosis transmembrane conductor (CFTR).

UPS occurs in all eukaryotes and has been well documented in different species of unicellular yeasts, fungi, plants, and mammals. In most cases, the cellular mechanisms underlying unconventional protein secretion are not well understood. Mechanistic underpinnings of UPS that have come to light in recent years range from facilitated cytoplasm-extracellular translocation through the PM [4] to processes that generate PM-fusing secretory autophagosomes [5], secretory lysosomes, or extracellular vesicles such as exosomes and ectosomes [6]. In terms of function, unconventional secretion is known to play diverse and important roles during embryonic development, in the regulation of cellular/systemic homeostasis and signaling, as well as in disease pathology and progression. The special issue in Cells entitled ‘Unconventional protein secretion in development and disease’ has included in its collection an original article and three review articles that cover UPS from different perspectives, all of which bear some connections with human diseases (https://www.mdpi.com/journal/cells/special_issues/UPS#keywords).

Miura and Ueda [7] reviewed UPS in yeast and fungi, with a particular focus on UPS evaluation by fungal secretome analysis. The authors discussed several major examples of unconventionally secreted yeast and fungal products, including the very well-studied Acyl-CoA-binding protein 1 (Acb1) [8,9] that is secreted by an autophagosome-dependent process and for which a specialized compartment for unconventional secretion (CUPS) has been identified morphologically in yeast [10]. The authors also elaborated on how a host of fungal protein allergens that are polypeptides lacking an ER-targeting signal peptide and are secreted unconventionally, are identified based on secretory proteomics analyses. Given that these allergen molecules are also found in yeast, analysis of the cellular mechanism underlying their unconventional secretion could be facilitated by the very established yeast genetics.

Lee and Ye [11] provided a comprehensive view of the biology of UPS processes in mammalian cells and focused on the role of endos-lysosomal compartments in cellular homeostasis via UPS. Classically known to function in endocytosis and not secretion, the endo-lysosomal compartments could nonetheless also act as exocytic or secretory compartments. The authors discussed their earlier work on the process of misfolding-associated protein secretion (MAPS) [12] and drew comparisons with the more commonly known process of chaperone-mediated autophagy, both of which involve chaperone-mediated lysosomal membrane translocation. It was postulated that MAPS or its dysfunction might be linked to proteostasis perturbation in neurodegenerative diseases, such as ceroid lipofuscinosis and Parkinson’s disease. The authors highlighted pressing questions in the field, including how cytosolic proteins are selected explicitly for secretion via late endosomes, as well as whether there is a specialized subpopulation of late endosomal compartments that is dedicated to protein secretion as oppose to endocytic traffic.

Mutations in the multi-membrane spanning chloride channel CFTR underlies the hereditary disease Cystic Fibrosis (CS) [13], with affected patients producing abnormally thick mucus that blocks ducts, intestines, and the bronchi. The most common form of CFTR mutation in CS patients is F508Δ, and the mutant proteins ER exit and PM transport are checked and retained by the ER quality control (ERQC) mechanism, leading to its degradation. However, a small fraction of F508Δ could be transported to the PM by both conventional transport and, more interestingly, by autophagy-dependent UPS [14]. Santos and colleagues [15] presented a comprehensive analysis of CFTR’s ER exit by interaction proteomics and bioinformatics to characterize proteins that interact with either selected CFTR peptide motifs or CFTR variants, including proteins that are either retained by or could escape the ERQC checkpoints to a varying degree. Based on the analyses, the authors concluded that the folding status of the CFTR mutants and variants is key in determining the CFTR interactome, which is recognized by the ERQC checkpoint. The work adds to our understanding of how CFTR’s ability to interact with other proteins mediates its ER exit and PM transport. 

Huntington’s disease (HD) is a hereditary neurodegenerative disorder resulting from a trinucleotide CAG (polyglutamine) repeat extension of the protein Huntingtin (HTT) [16]. HD neuropathology is mainly due to a toxic gain-of-function by mutant *HTT* (mHTT) and its exon 1 coded proteolytic products, which form both nuclear and cytoplasmic aggregates that perturb neuronal processes ranging from gene transcription to axonal transport. Tang [17] reviewed how neurons could secrete cytoplasmic mHTT via UPS [18]. Interestingly, mHTT could also be transported between neuronal cells by actin-based somatic connections known as tunneling nanotubes (TNTs) [19]. These unconventional modes of pathological protein transmission could allow mHTT and its aggregates in a diseased neuron to spread to neurons at its proximity and those that are connected within neural circuits, thereby potentiating non-cell autonomous pathology of mHTT.

Our understanding of conventional secretion and its underlying mechanisms represents a pivotal foundation to our contemporary view of cellular dynamics and physiology and is invaluable in advancing knowledge on human disease mechanisms and their treatment. Even from this small collection of papers, it could be gleaned that UPS has pathophysiological roles that could be just as important. Future work that would provide a better understanding of the cellular mechanisms of various modes of UPS would certainly change our current picture on cellular dynamics, and likely provide more therapeutic handles to human diseases.
